# Development of High Sensitivity Humidity Sensor Based on Gray TiO_2_/SrTiO_3_ Composite

**DOI:** 10.3390/s17061310

**Published:** 2017-06-07

**Authors:** Min Zhang, Shunhang Wei, Wei Ren, Rong Wu

**Affiliations:** 1 The School of Physics Science and Technology, Xinjiang University, Urumqi 830046, China; minzhang0816@163.com; 2 Shanghai Key Lab of Chemical Assessment and Sustainability, School of Chemical Science and Engineering, Tongji University, 1239 Siping Road, Shanghai 200092, China; shun_hang@126.com; 3 Xinjiang Key Laboratory of Electronic Information Materials and Devices, Xinjiang Technical Institute of Physics & Chemistry, CAS, Urumqi 830011, China

**Keywords:** humidity sensor, gray TiO_2_/SrTiO_3_ composite, semiconductor

## Abstract

A gray TiO_2_/SrTiO_3_ composite nanocrystalline sensor with narrow band-gap was successfully prepared through a facile wet chemical method. The precursor was calcined in N_2_ flow under atmospheric pressure and thereafter, a humidity sensor based on the composite was fabricated. The sensor showed high resistive sensitivity and varied by more than four orders of magnitude with an increase in relative humidity (RH) from 11% to 95%. The response and recovery time were about 3.1 s and 76 s, respectively with maximum hysteresis at 1% RH. In comparison with pure SrTiO_3_ and black TiO_2_, the gray composite based device exhibits a higher sensitivity. These results demonstrate the potential of gray TiO_2_/SrTiO_3_ for humidity sensing applications.

## 1. Introduction

Humidity sensors play a crucial role in environment control, food science, plant cultivation, and the medical field [1]. In 1996–1998, high sensitivity humidity sensors using quartz oscillator and open capacitor were reported, which have advantages of fast response time, immunity to electromagnetic interference and high temperature stability. Besides, the differential sensors made of ceramic material are low-cost and required for high precision measurements [2,3]. Currently, the humidity sensing materials include semiconductors, perovskite oxides, and polymers [4,5,6,7]. Among these materials, sensors based on perovskite oxides are of high reliability and sensitivity. Some perovskite-type materials such as NaTaO_3_, BaTiO_3_ and LaFeO_3_ have been reported in the literature for their humidity detecting applications [6,8,9].

SrTiO_3_ is recognized as one of the most promising perovskite oxides for sensing applications. Its wide band gap and large exciton binding energy induce excellent electrical and optoelectronic properties [10], owing to which the material conducts electrically at room temperature through the adsorption of water molecules on a surface. Interestingly, there has been limited research work performed on SrTiO_3_ based sensors and the primary focus has been on the conventional white SrTiO_3_ (band-gap 3.2 eV for bulk material).

However, to the best of our knowledge, the sensing properties of dark colored SrTiO_3_ such as humidity sensing, gas sensing, or photo-sensing characteristics have never been reported. Some research work regarding dark colored semiconductors was published by X. Chen in 2011. The black TiO_2_ with a narrow band-gap of 2.18 eV shows great solar absorption for photocatalysis application [11]. Thereafter, the black TiO_2_ has attracted considerable attention due to their enhanced photoelectric properties [12]. In the current work, gray TiO_2_/SrTiO_3_ composite with a narrow band-gap was successfully prepared by a facile one-step synthetic method. The gray composite exhibits better humidity sensing performance in comparison with pure SrTiO_3_. Moreover, a narrow band-gap would be more favorable for generation of excited carriers and an increase in conductivity. Hence, sensors based on the dark colored semiconductors might possess more flexibility in realization of high performance multifunctional sensors, which endows composites with wider applications. 

In the current work, gray TiO_2_/SrTiO_3_ composite nanocrystalline was synthesized in order to be used as a sensing material. Thereafter, the humidity sensor was fabricated by coating the material on carbon interdigitated electrodes. The humidity sensing characteristics were studied by measuring the impedance at different relative humidity (RH). The device shows excellent sensitivity, good linearity, and small hysteresis, which demonstrates that gray TiO_2_/SrTiO_3_ composite is a suitable candidate for humidity sensors.

## 2. Experimental Procedure

In the experiment, 14 mL tetrabutyl titanate was dissolved in 20 mL ethanol. Then, 1 g urea (used for N doping) was dropped to obtain solution A, whereas solution B contained 5 mL deionized water, 10 mL ethanol, and 1 mL hydrochloric acid. Thereafter, solution B was gradually added dropwise to solution A under stirring with a glass rod until a white colloid was formed. Thereafter, colloid was kept in a water bath heated at 35 °C for half an hour. Subsequently, equimolar Sr(NO_3_)_3_ (Sr:Ti = 1:1) was added into the mixture under magnetical stirring. The resulting material was calcined in N_2_ flow under atmospheric pressure at 500–800 °C. The material synthesized at low temperature was TiO_2_/SrTiO_3_ composite; while that obtained at high temperature was more apt to form pure SrTiO_3_. The synthesized sample was mixed with deionized water in a weight ratio of 5:1 to form a paste. The paste was coated on a ceramic substrate (1.0 cm × 0.5 cm) with four pairs of carbon interdigitated electrodes to form a sensitive film. The film was dried in vacuum at 60 °C. The preparation details of black TiO_2_ are given elsewhere [12].

The crystal structure of the sample was examined by X-ray diffraction (XRD, Bruker, D8 Advance, Karlsruhe, Germany). The optoelectronic characterization of the films was examined by PerkinElmer UV/Vis spectrometer (Lambda 650 S, Waltham, MA, USA) at room temperature while Scanning electron microscope (SEM) (LEO1430VP, Zeiss, Jena, Germany) was used to investigate the morphology. The electrical properties of the sensor were measured by a Precision Impedance Analyzer 6500B Serious from Wayne Kerr Electronics (London, UK). For the humidity sensing properties test, the sensor was put into six chambers with different RH values. The RH values ranging from 11 to 95% were obtained via using different saturated salt solutions as humidity generation sources. These saturated salt solutions were LiCl, MgCl_2_, Mg(NO_3_)_2_, NaCl, KCl, and KNO_3_ and the corresponding RH values were 11%, 33%, 54%, 75%, 85%, and 95%, respectively. The voltage was set at AC 1 V and the frequency changed from 40 Hz to 100 kHz in humidity studies. All the above device performance tests were operated at room temperature controlled by air conditioning.

## 3. Results and Discussion

**Figure 1:**
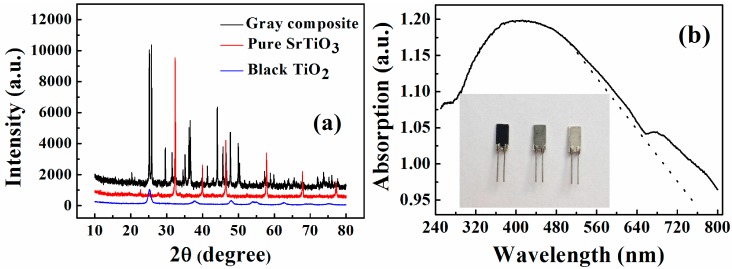
(**a**) X-ray diffraction (XRD) patterns of TiO_2_, TiO_2_/SrTiO_3_, and SrTiO_3_; (**b**) UV-Visible absorption spectrum of gray TiO_2_/SrTiO_3_ composite; Inset is the picture of humidity sensors based on different samples.

The X-ray diffraction (XRD) patterns of black TiO_2_, gray TiO_2_/SrTiO_3_ composite and pure SrTiO_3_ are shown in Figure 1a. The diffraction peaks of gray sample can be indexed to be peroviskite SrTiO_3_ and brookite TiO_2_ composite. The black sample corresponds to the black TiO_2_ with anatase type, and the third sample shows pure SrTiO_3_ with perovskite structure.

The dark color indicates the enhanced light absorption of the sample. The UV-visible absorption spectrum of gray TiO_2_/SrTiO_3_ composite is shown in Figure 1b. It is found that the absorption edge of the composite is approximately defined, which shows that absorption of the gray sample in visible light region is enhanced, in comparison with pure SrTiO_3_. According to the relationship between band-gap and absorption edge, Eg = 1240/λ (where Eg is the band-gap of semiconductor, λ is the light wavelength), the band gap of composite is estimated to be less than 2 eV [13]. These results demonstrate the potential of the composite in photo-sensitive applications. Inset is the picture of actual humidity sensors based on the three samples, respectively.

**Figure 2:**
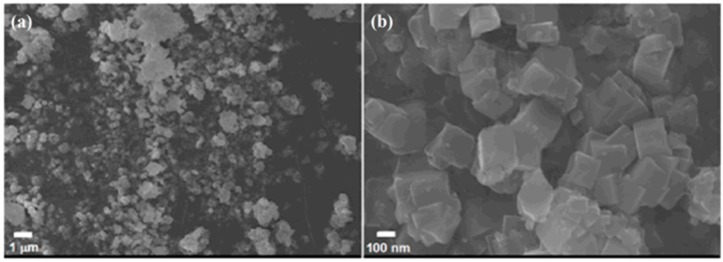
The SEM images of TiO_2_/SrTiO_3_ composite at low (**a**) and high (**b**) resolutions.

Figure 2a,b display the general morphology of the gray TiO_2_/SrTiO_3_ composite studied via SEM at different resolutions. It shows that the composite has a cube shape and the average size is about 150 nm. A uniform and crack-free TiO_2_/SrTiO_3_ sample is obtained, demonstrating that the composite is suitable to act as a substrate material for humidity sensors.

**Figure 3:**
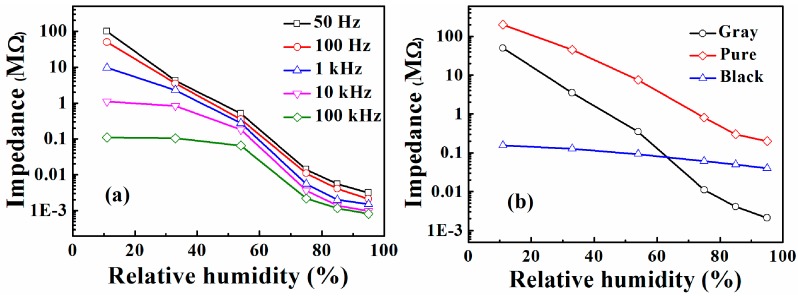
(**a**) Impedance versus relative humidity (RH) curves of TiO_2_/SrTiO_3_ humidity sensor at various frequencies; (**b**) The Impedance versus RH curves of sensors based on different samples.

The relationship between impedance and RH for TiO_2_/SrTiO_3_ humidity sensor at various frequencies is shown in Figure 3a. In the low frequency regions (50 Hz and 100 Hz), it can be seen that the impedance decreases with an increase in frequency and changes by far more than four orders of magnitude with a gradual increase in RH. The sensor presents a higher sensitivity compared with other ABO_3_-structured semiconductors reported previously, such as SrTiO_3_ nanospheres, NaTaO_3_, BaTiO_3_ [6,8,14]. The high sensitivity is closely related to the N-doping in composite, which induces more oxygen vacancies as an active site to adsorb more water molecules [12].

Additionally, at low frequencies, RH has a close-to-linear relationship with impedance, while there is no clear linearity of relationship at high frequencies. A high sensitivity and good linearity can be obtained at 100 Hz. Hence, 100 Hz was chosen as the ideal working frequency. Moreover, the impedance of referent ceramic substrate remained constant w.r.t change in RH, which signifies that the substrate has no contribution in humidity sensing.

Figure 3b shows the impedance versus RH curves of sensors based on different samples at 100 Hz. The sensitivity of pure SrTiO_3_ device changes by about three orders of magnitude while that of black TiO_2_ changes within one order of magnitude. The gray sample-based sensor exhibited a much higher sensitivity in comparison with that of black TiO_2_ and pure SrTiO_3_. The composites composed of different materials usually combine advantages of the components with better properties. For example, In_2_O_3_-NiO, ZnO-In_2_O_3_ and MoO_3_-NiO composites all exhibited enhanced humidity sensitive characteristics [5,15,16]. The strengthened moisture properties may be attributed to the improved specific surface area and more active sites in composite. A large surface area-to-volume ratio means that a significant fraction of the atoms or molecules are surface atoms that can participate in surface reactions [15,16].

**Figure 4:**
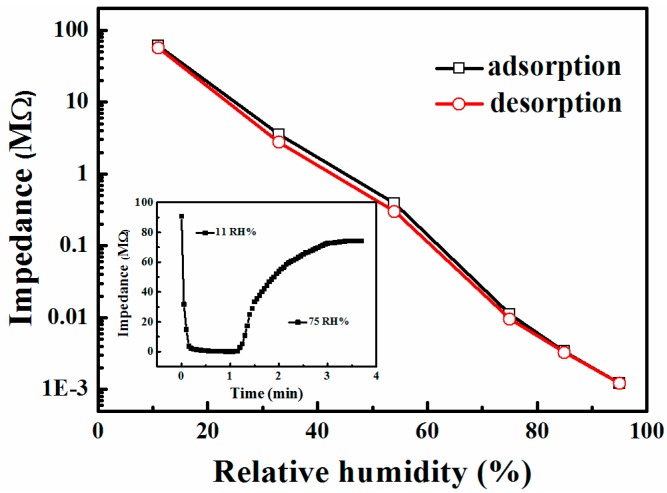
Humidity hysteresis characteristics of TiO_2_/SrTiO_3_ sensor measured at 100 Hz and AC 1 V; Inset shows the response and recovery behavior of TiO_2_/SrTiO_3_ humidity sensor.

Figure 4 shows the humidity hysteresis characteristic of the composites between the adsorption and desorption process at 100 Hz. The TiO_2_/SrTiO_3_ sensor appeared to have a narrow hysteresis loop. The maximum hysteresis of the device was about 1% RH, which was smaller than that of the pure SrTiO_3_ (less than 2% RH as measured).

The response time is the time taken by a sensor to achieve 90% of the total resistance change in the case of adsorption process or the recovery time in the case of desorption process. Since the humidity detection range primarily covers the range of 11% RH–75% RH, the response and recovery characteristics of the sensor in this range were measured at 100 Hz, as shown in the inset. The response time for the change from 11% RH to 75% RH was about 3.1 s, and recovery time for the change from 75% RH to 11% RH was about 76 s.

**Figure 5:**
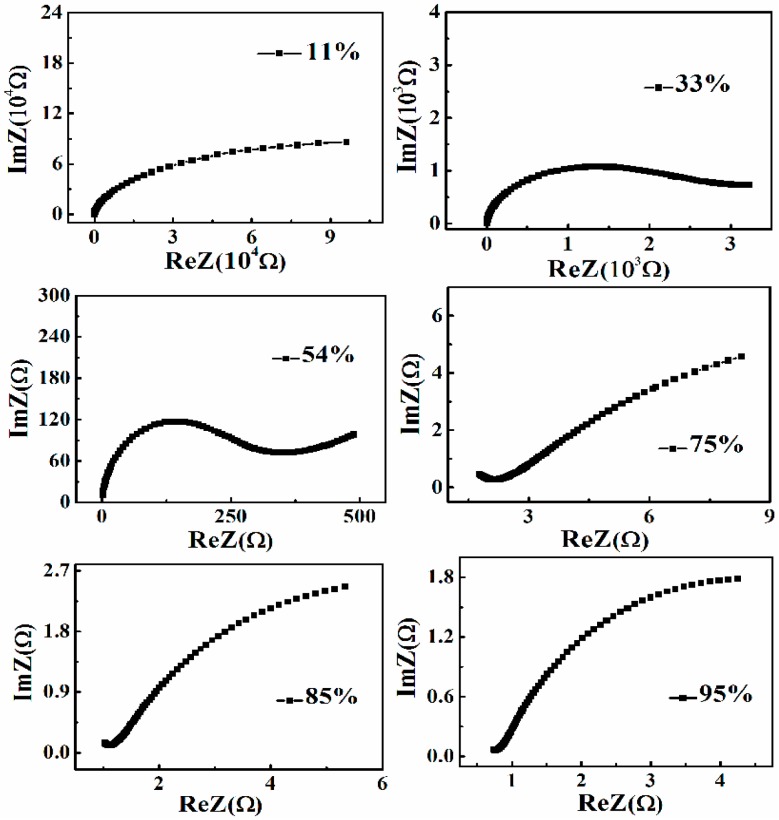
Complex impedance properties of the composite measured at different RH.

To have an enhanced understanding of the sensing mechanism, the complex impedance plots of TiO_2_/SrTiO_3_ sensor at different RH are presented in Figure 5 for the frequency range of 40 KHz to 100 kHz. At low RH, only a few water molecules are adsorbed by chemisorption on the active site of the composite surface to form hydroxyl groups. Subsequently, protons would be transferred from the surface hydroxyl groups to water molecules to form hydronium ions (H_3_O^+^) [6]. Though the density of H_3_O^+^ is low and the transfer of carriers is difficult, the charge carriers would bring about electrons accumulation and bend the energy-band at the grain surface, leading to a decreased resistance. Meanwhile, bounded electrons emerge by means of polarization of the sensing material. At this time, the dominant conducting carriers are H_3_O^+^ and bounded electrons [17].

Starting from the spectrum of 54% RH, an evident straight line in the low frequency region appears which is the consequence of diffusion and polarization of charged carriers between sensing film and electrodes [18]. With the RH increase, many more water molecules are adsorbed on the surface and tend to form a liquid-like layer, in which a proton transfers between neighboring water molecules. Hence, protons become dominant charged carriers at high RH. Correspondingly, the semicircle in the high frequency region fades away and the straight line in the low frequency region becomes very obvious.

It is well known that the characteristic frequency corresponds to the value when the imaginary part of the complex impedance spectrum reaches its maximum, near which is known as the dispersion region. Seen from the spectra in Figure 5, the dispersion region gradually moves toward high frequency as the RH increases, which reveals that the space-charge polarization does exist in the sensor. Based on the research proposed by Paula Vilarinho et al., the dielectric constant of SrTiO_3_ increases as the frequency increases, then the polarization of adsorbed water molecules is enhanced with increasing RH [19].

## 4. Conclusions

In conclusion, the synthesis, characterization, and humidity sensing of TiO_2_/SrTiO_3_ were investigated. The impedance changes by more than four orders of magnitude in the range from 11% to 95% RH at 100 Hz, and the maximum hysteresis is about 1% RH. Furthermore, the sensing mechanism was discussed by the complex impedance spectra in detail. These results demonstrate the potential application of the composite in fabricating high performance humidity sensors.
